# The Ontario Pharmacy Evidence Network Interactive Atlas of Professional Pharmacist Services

**DOI:** 10.1177/17151635211004969

**Published:** 2021-05-28

**Authors:** Suzanne M. Cadarette, Nancy He, Maha Chaudhry, Lisa Dolovich

**Affiliations:** Leslie Dan Faculty of Pharmacy, University of Toronto, Toronto, Ontario; Dalla Lana School of Public Health, University of Toronto, Toronto, Ontario; ICES, Toronto, Ontario, University of Waterloo, Kitchener, Ontario; Eshelman School of Pharmacy, University of North Carolina at Chapel Hill, North Carolina, United States; Leslie Dan Faculty of Pharmacy, University of Toronto, Toronto, Ontario; ICES, Toronto, Ontario, University of Waterloo, Kitchener, Ontario; Leslie Dan Faculty of Pharmacy, University of Toronto, Toronto, Ontario; Leslie Dan Faculty of Pharmacy, University of Toronto, Toronto, Ontario; School of Pharmacy, University of Waterloo, Kitchener, Ontario; Department of Family Medicine, McMaster University, Hamilton, Ontario, University of North Carolina at Chapel Hill, North Carolina, United States

## Introduction

**Figure 1:**
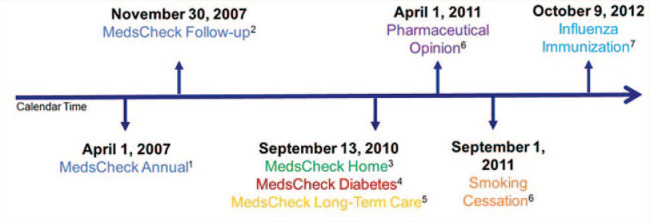
Timeline of the availability of publicly funded professional community pharmacy services in Ontario Program eligibility as of July 2020, in addition to being a resident with a valid Ontario health card: ^1^Taking 3 or more medications for chronic conditions. ^2^Received a MedsCheck and require follow-up consultation (hospital discharge, pharmacist documented decision, physician or nurse practitioner referral, planned hospital admission). ^3^Eligible for community MedsCheck services yet are unable to visit a community pharmacy in person. ^4^Living with type 1 or type 2 diabetes. ^5^Living in a licensed long-term care home; program delisted January 1, 2020.^27^ ^6^Ontario Drug Benefit program eligible recipients and persons younger than 25 without private drug insurance. ^7^Aged 5 or more years.

Over the past few decades, the role and scope of pharmacists in Canada has broadened to provide a more effective platform upon which to contribute to outcomes-driven medication management.^1^ Community pharmacists are among the most accessible health care providers within the community-based health care system and have offered a growing list of professional pharmacy services as a consequence of professional evolution.^1,2^ Since 2007, the government of Ontario has leveraged community pharmacist expertise in medication management by introducing and remunerating community pharmacies for the following professional pharmacist services: medication reviews through MedsCheck programs (Annual, Diabetes, Home, Long-Term Care),^3-6^ communicating with prescribers regarding drug therapy-related problems (Pharmaceutical Opinion program),^7,8^ providing smoking cessation counselling services (pharmacy smoking cessation program)^9^ and administering influenza immunizations^10,11^ (Figure 1). Pharmacies submit claims to the Ontario government through the Ontario Drug Benefit program for renumeration for each service (Table 1). We received funding from the Government of Ontario as part of the Ontario Pharmacy Evidence Network (OPEN) program peer-reviewed Health Service Research Fund to complete descriptive analyses of professional pharmacy services delivery across the province. These analyses are introduced here as the OPEN Interactive Atlas of Professional Pharmacist Services (Box 1).^12^ This research brief provides an overview with technical detail to support the Atlas.^12^ Future briefs will summarize each service separately.

**Table 1:** Summary of pharmacy service billing codes, immunization codes, and service fees

Service	Product Identification Number (PIN)	Fee*
MedsCheck	93899979	$60
Follow-up: Hospital discharge	93899981	$25
Follow-up: Pharmacist documented decision	93899982	$25
Follow-up: Physician/nurse practitioner referral	93899983	$25
Follow-up: Planned hospital admission	93989984	$25
MedsCheck Diabetes	93899988	$75
Follow-up	93899989	$25
MedsCheck Home	93899987	$150
MedsCheck Long-Term Care**	93899985	$90
Follow-up: Quarterly**	93899986	$50
Pharmaceutical Opinion: Not filled	93899991	$15
Pharmaceutical Opinion: No change	93899992	$15
Pharmaceutical Opinion: Change	93899993	$15
Smoking Cessation First Consultation	93899941	$40
Follow-up: Primary	93899942	$15
Follow-up: Secondary	93899943	$10
Quit status: Successful	93899944	$0
Quit status: Unsuccessful	93899945	$0
Quit status: Unknown	93899946	$0
Service	Drug Identification Number (DIN)	Fee
Influenza Immunization: Injectable***	02346850, 02223929, 02015986, 02362384, 09857501, 02428881, 02420686, 02269562, 02420783, 02432730, 02420642, 02420643, 02473283, 02473283	$7.50
Influenza Immunization: Nasal***	02426544	$5.00

Table is colour coded to coincide with service-specific colours used to represent each type of service in the Atlas interactive tool.

* Current as of July 2020; MedsCheck Annual was $50 from April 2007 to September 2016.

** MedsCheck Long-Term Care services were delisted effective January 1, 2020.^27^

*** Includes eligible DINs as of the 2019/2020 influenza season; nasal was only available in the 2015/2016 to 2018/2019 influenza seasons.

**Box 1:** Examples of how to leverage the OPEN Atlas interactive tool

This research brief serves to introduce the interactive OPEN Atlas Tool with some key examples and methodological detail. Some examples of how to leverage the tool are provided here: • Trends in the uptake of new pharmacy services or following policy changes in Ontario can be used to help inform planning for the delivery of new or modified pharmacist services in other provinces and territories. • Regional differences identified in Ontario can identify target areas for future research to better understand program barriers and facilitators. • Age group and sex-specific rates can help identify potential gaps in service delivery for different patient groups for targeted intervention. Future briefs will present and interpret results by service (influenza immunization, MedsCheck, Pharmaceutical Opinion, smoking cessation) and provide context across Canada.

## Methods

### Overview and indicators of professional pharmacist services

A health care atlas provides summaries of trends and regional differences in indicators of health or health care delivery.^13^ Online descriptive tools that display large-scale health care administrative data have recently become available that permit interactive manipulation of key descriptive factors such as region, calendar year, sex and age groups.^14-16^ Our aim was to create an interactive atlas of professional pharmacist services in Ontario (OPEN Interactive Atlas Tool). We consulted with the OPEN Advisory Committee and staff of the Ontario College of Pharmacists to identify and provide feedback on relevant indicators. Three main indicators were chosen and are represented in the interactive atlas tool as distinct tabs for each service:

1) **Trends:** trends in number of monthly claims
2) **Age and sex:** crude counts and rates by age group and sex
3) **Maps:** choropleth maps representing regional summaries of standardized rates

Each tab includes the ability to manipulate results based on a priori defined characteristics, such as region, sex and calendar year or influenza season. Given the similarities in formatting and presentation of results across each service, we strategically used different colours to represent each service as presented in Table 1—original MedsCheck (Annual): blue; MedsCheck Diabetes: red; MedsCheck Home: green; MedsCheck Long-Term Care: yellow; Pharmaceutical Opinion: purple; Smoking Cessation: orange; and Influenza Immunization: light blue.

### Data sources

We accessed 2 health care administrative databases housed at ICES for this research: 1) *Ontario Drug Benefit Database* to identify professional pharmacist service claims billed from program launch to December 31, 2019, or to March 31, 2020, for influenza immunizations and 2) *Registered Persons Database* to capture demographic information (age, sex, postal code) for residents receiving pharmacist services. These data sets were linked using unique encoded identifiers and analyzed at ICES. Regional analyses were based on patients’ Local Health Integration Network (LHIN) at the time of first service delivery in each calendar year or influenza season. LHINs have been responsible for supporting the integration of health care at the local level, such as hospitals, community health centres and long-term care homes, since 2007.^17^ In spring 2019, the Ontario government began to consolidate Ontario’s LHINs with 6 provincial health agencies to form the super-agency Ontario Health in an effort to improve efficiencies.^18,19^ This merging process was intended to be finalized in April 2020; however, it has been on hold as of March 2020 due to the coronavirus disease (COVID-19) pandemic.^20^ Therefore, local health care delivery structures through LHINs were in place over the entire study period, yet regional groupings will be reconsidered as relevant in future.

We used 2016 Statistics Canada intercensal estimates and 2018 regional boundaries to obtain annual population estimates for Ontario by age group, sex and region grouped by LHIN.^21^ The 2018 estimates were used for the 2019 calendar year and 2019/2020 influenza season, since these data were the most recent at the time of analysis. We used Statistics Canada’s 2011 Health Region Boundary spatial file to create regional boundaries in maps of service delivery.^22^

### Cohort creation and exclusion

We created cohorts of Ontario residents accessing each professional pharmacist service based on the date of first service claim submitted to the Ontario Drug Benefit plan, by type of service. For each cohort, patients were excluded due to the following data errors: missing age or sex and death date before first service date. Patients with missing postal code were only excluded from regional analyses.

### Analyses

**Figure 2:**
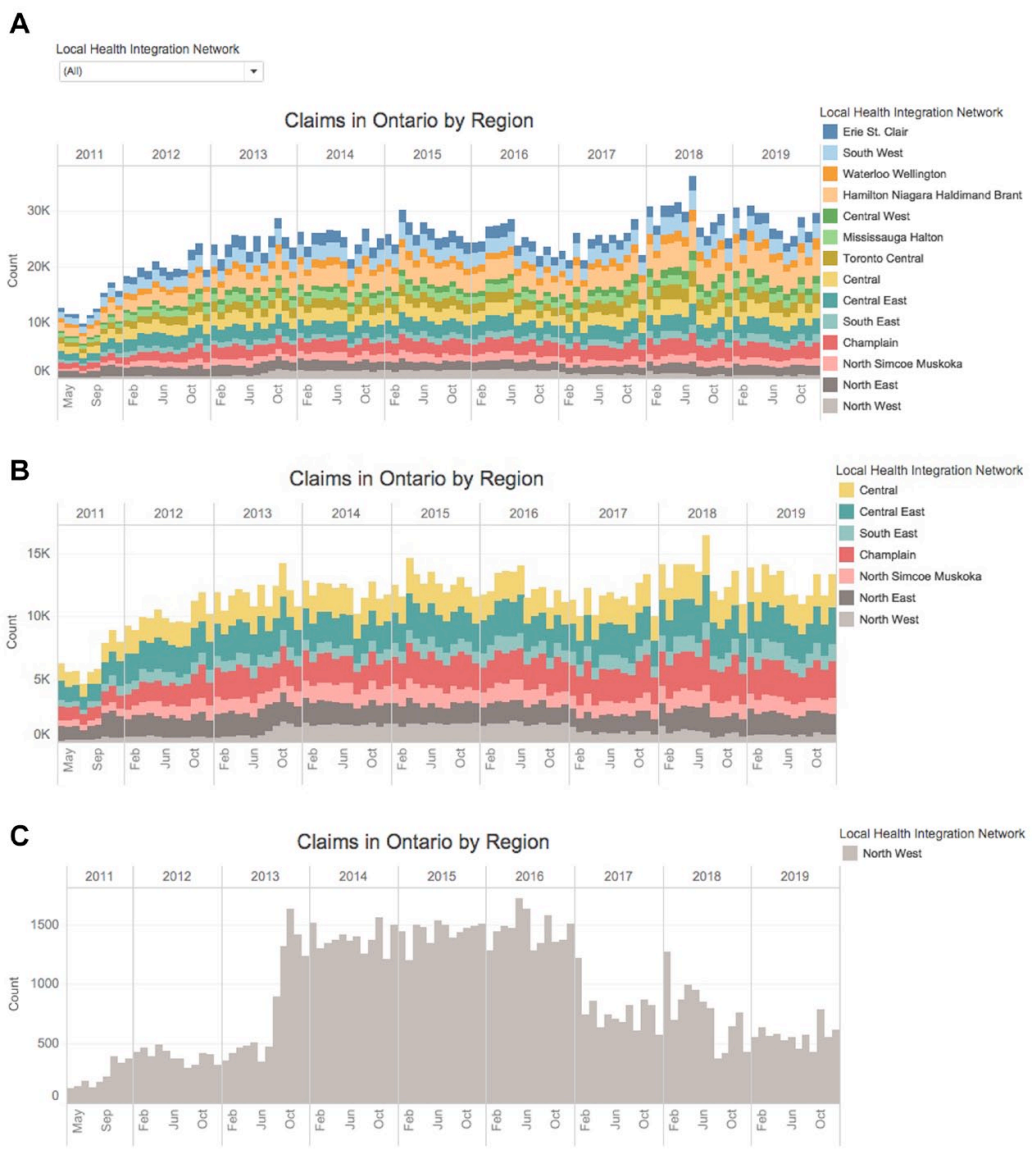
Number of Pharmaceutical Opinion services claimed by month and Local Health Integration Network (LHIN). (A) All 14 LHINs, (B) 7 north and east LHINs, and (C) only northwest region

*Trends Tab*: The monthly number of claims are presented for each service as 2 histograms: 1) interactive by region (LHIN) and 2) interactive by type of service within each professional program (grouped as depicted by colour coding in Table 1). Figure 2 provides an example for an interactive histogram by LHIN for the Pharmaceutical Opinion program. Panel A includes all regions (14 LHINs), panel B includes half of the regions (7 most eastern and northern LHINs) and panel C only includes the northwest LHIN. As is apparent in the figure, the interactive tool automatically reformats the y-axis to facilitate interpretation based on the maximum relevant number of monthly services; this includes a maximum of 40,000 when all regions are included, yet only 16,000 when restricted to 7 regions and 1800 when restricted to the northwest LHIN. The benefits of considering trends by individual region also become clear in our example. Whereas a relatively stable number of claims, or perhaps slight increase from 2014 to the end of 2019, is visually apparent when considering all or half of the LHINs, a clear drop and decline in the number of claims after 2016 is apparent when restricted to the northwest LHIN.

**Figure 3:**
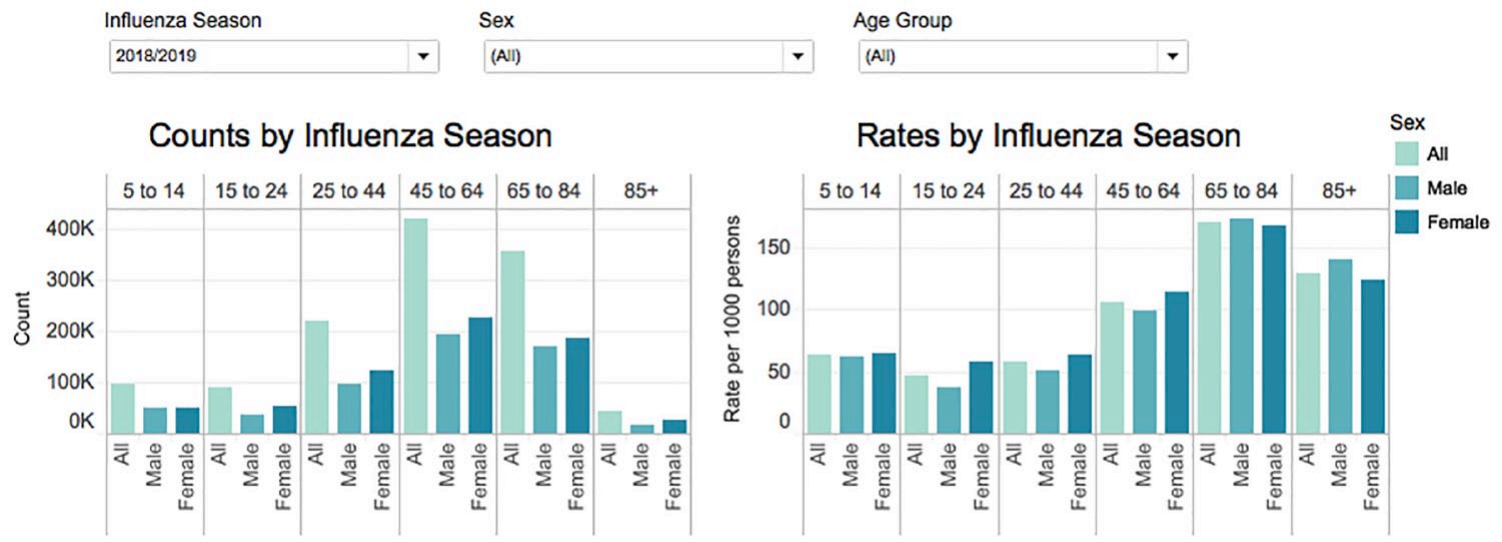
Crude counts and rates per 1000 persons by age group and sex for the 2018/2019 influenza season

*Age and Sex Tab*: Crude counts and rates of service delivery are presented as interactive histograms by 1) calendar year or influenza season and 2) age groupings. Age was categorized into groups based on program eligibility and consultation with the OPEN Advisory Committee (Appendix 1, available online at www.cpjournal.ca). Rates were calculated per 10,000 persons for pharmacist smoking cessation services and 1000 persons for all other services (Appendix 1).^23^ Counts are important to understand overall delivery, yet rates help compare use between sexes after adjusting for population size. For example, Figure 3 illustrates that more women than men aged 65 or more years received influenza immunizations in the 2018/2019 season, yet immunization rates are higher among older men (e.g., 140.6/1000 men and 123.4/1000 women aged 85 or more years).

The Age and Sex Tab also presents the overall age distribution as histograms for each service based on the age at first service delivery. Summary descriptive statistics (mean, standard deviation, median and interquartile bounds) are provided overall and by sex in a table.

**Figure 4:**
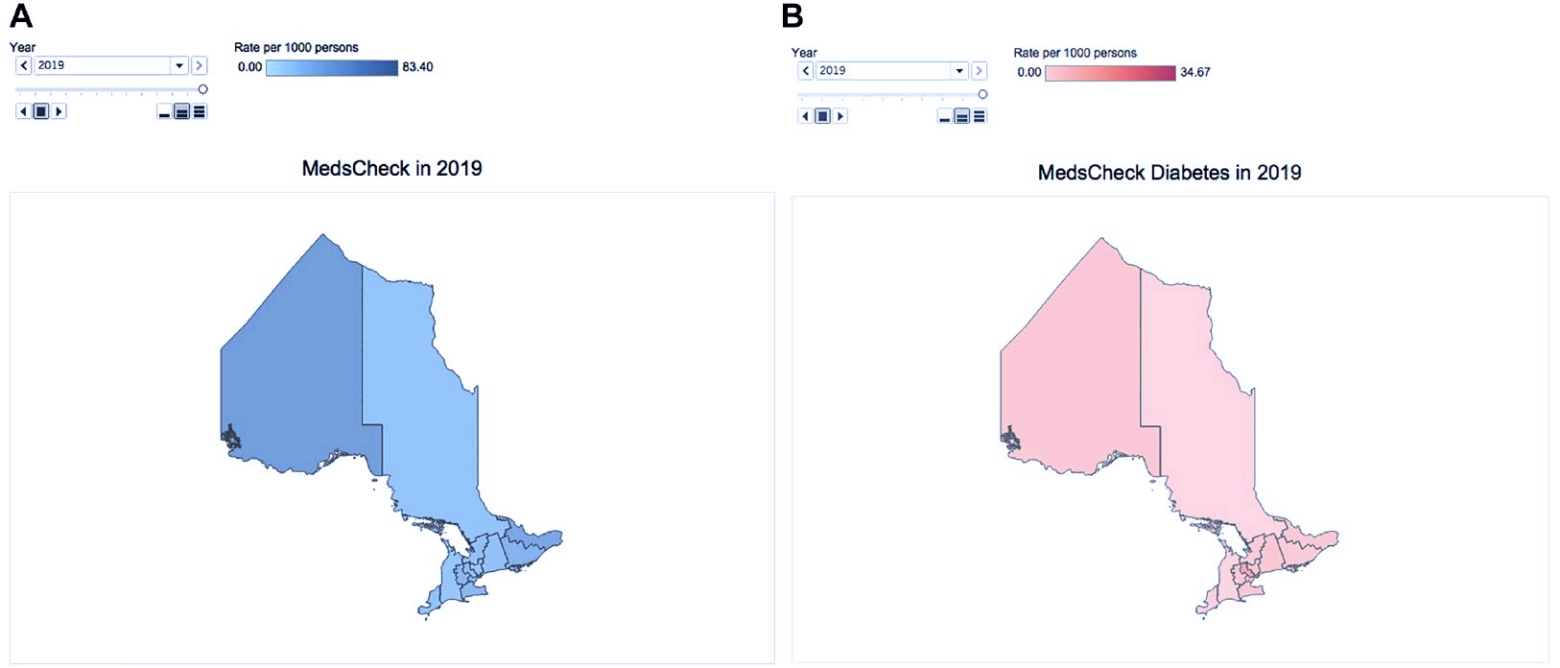
Samples of choropleth maps of age group and sex-standardized rates of services for 2019 calendar year. (A) MedsCheck and (B) MedsCheck Diabetes Deeper colour represents higher age and sex-standardized rates of service delivery.

*Maps Tab*: Age group and sex-standardized rates by LHIN are presented by year or influenza season as choropleth maps. The scale range is from 0 to the maximum age-sex standardized rate of that pharmacist service, with deeper colour density reflective of higher service delivery rates. Figure 4 includes examples of the static maps for the 2019 calendar year. Regional differences are clearly visible across services based on colour density. The interactive Atlas Tool provides the ability to click forward or backward by calendar year or influenza season. In addition, a separate button is included that, when selected, plays a video loop of the change in regional service delivery over time by calendar year or influenza season.

### Software and small cell considerations

We used SAS Enterprise Guide 7.1 (SAS Institute, Cary, North Carolina)^24^ to complete analyses of health care administrative data at ICES, Microsoft Excel 2016 (Microsoft Corporation, Redmond, Washington)^25^ to calculate crude and age group/sex-standardized rates and Tableau Desktop Professional Edition version 2018.3.16 (Tableau Software, Seattle, Washington)^26^ to create the interactive atlas tool. ICES data-sharing agreements restrict reporting small cells (cell counts <6); thus, small cells and cells that could be used to back-calculate them were reported as null in all figures.

## Discussion

The purpose of this research brief is to supplement the interactive OPEN Atlas Tool by providing details on the methods used to derive the data shown in the Tool. Future briefs in this series will summarize each service separately, providing a snapshot of services in Ontario with interpretation of the data and a summary of the availability of similar services across Canada. We encourage other provinces and territories to consider creating similar descriptive atlases of pharmacist services as a starting point for discussion, collaboration and education. As community pharmacy practice evolves across Canada and we learn to pivot to the changing health care delivery afforded by external factors such as the COVID-19 pandemic, more ready access to descriptive tools such as the OPEN Atlas of Professional Pharmacist Services will become a helpful input into pharmacy and broader health services planning.

## Supplemental Material

sj-pdf-1-cph-10.1177_17151635211004969 – Supplemental material for The Ontario Pharmacy Evidence Network Interactive Atlas of Professional Pharmacist Services Click here for additional data file. Supplemental material, sj-pdf-1-cph-10.1177_17151635211004969 for The Ontario Pharmacy Evidence Network Interactive Atlas of Professional Pharmacist Services by Suzanne M. Cadarette, Nancy He, Maha Chaudhry and Lisa Dolovich in Canadian Pharmacists Journal / Revue des Pharmaciens du Canada
