# Supplementary material for: The Ontario Pharmacy Evidence Network Interactive Atlas of Professional Pharmacist Services
Source: Can Pharm J (Ott). 2021 May 28;154(3):153–9. doi: 10.1177/17151635211004969 (PMC8165887; doi:10.1177/17151635211004969)
Supplement: sj-pdf-1-cph-10.1177_17151635211004969 – Supplemental material for The Ontario Pharmacy Evidence Network Interactive Atlas of Professional Pharmacist Services [file sj-pdf-1-cph-10.1177_17151635211004969.pdf]

APPENDIX 1

Age groupings by type of service

	Influenza Immunization	MedsCheck and Pharmaceutical Opinion	Smoking Cessation
Age groups (years)*	5-14, 15-24, 25-44<br>45-64<br>65-84<br>85+	<45<br>45-64<br>65-84<br>85+	12-44<br>45-64<br>65+

*Age based on first date of service in the calendar year or influenza season. Age groups were determined upon consultation with the Ontario Pharmacy Evidence Network (OPEN) Advisory Committee. Persons aged <5 (influenza immunization), <12 (smoking cessation), <18 MedsCheck Long-Term Care, or <0 (other MedsCheck services and Pharmaceutical Opinion services) were deemed errors and deleted before analyses began.

Example rate calculations

Age-group-specific rate by LHIN $= E_i = \left(\frac{e_{i\,(f)}}{p_{i(f)}} * P_{i(f)}\right) + \left(\frac{e_{i\,(m)}}{p_{i(m)}} * P_{i(m)}\right)$

Age-group/sex-standardized rate by LHIN $= \frac{\sum E_i}{\sum P_i} * Multiplier$

$e_{i(f)}$ = the number of females receiving service in age group i for a specific LHIN
$e_{i(m)}$ = the number of males receiving service in age group i for a specific LHIN
$p_{i(f)}$ = the number of females in age group i for a specific LHIN
$p_{i(m)}$ = the number of males in age group i for a specific LHIN
$P_{i(f)}$ = the number of females in age group i for the Standard Population
$P_{i(m)}$ = the number of males in age group i for the Standard Population
P_i = the number of people in age group i for the Standard Population

Multiplier = Multiply by 10,000 for Smoking Cessation service and 1,000 for all other services.

Equations

Example equations to calculate age-group specific and age-group/sex-standardized rates of service delivery by region defined by Local Health Integration Network (LHIN) for a given year or influenza season that are depicted in the Maps Tab. Simpler equations were used for age-group and sex-specific rates in the Age and Sex Tab. For example, when calculating age-group specific rates for males, only males were included in each equation.

Cadarette SM, et al. The Ontario Pharmacy Evidence Network Interactive Atlas of Professional Pharmacist Services. *Can Pharm J (Ott)* 2021;154. DOI: 10.1177/17151635211004969.
